# Activated, Pro-Inflammatory Th1, Th17, and Memory CD4+ T Cells and B Cells Are Involved in Delayed-Type Hypersensitivity Arthritis (DTHA) Inflammation and Paw Swelling in Mice

**DOI:** 10.3389/fimmu.2021.689057

**Published:** 2021-08-02

**Authors:** Gaoyang Li, Shrikant Shantilal Kolan, Shuai Guo, Katarzyna Marciniak, Pratibha Kolan, Giulia Malachin, Franco Grimolizzi, Guttorm Haraldsen, Bjørn Steen Skålhegg

**Affiliations:** ^1^Department of Nutrition, Institute of Basic Medical Sciences, University of Oslo, Oslo, Norway; ^2^Department of Pathology, Oslo University Hospital and University of Oslo, Oslo, Norway

**Keywords:** CD4+ T cell, methotrexate, rheumatoid arthritis, DTHA mouse model, dexamethasone

## Abstract

Delayed-type hypersensitivity arthritis (DTHA) is a recently established experimental model of rheumatoid arthritis (RA) in mice with pharmacological values. Despite an indispensable role of CD4+ T cells in inducing DTHA, a potential role for CD4+ T cell subsets is lacking. Here we have quantified CD4+ subsets during DTHA development and found that levels of activated, pro-inflammatory Th1, Th17, and memory CD4+ T cells in draining lymph nodes were increased with differential dynamic patterns after DTHA induction. Moreover, according to B-cell depletion experiments, it has been suggested that this cell type is not involved in DTHA. We show that DTHA is associated with increased levels of B cells in draining lymph nodes accompanied by increased levels of circulating IgG. Finally, using the anti-rheumatoid agents, methotrexate (MTX) and the anti-inflammatory drug dexamethasone (DEX), we show that MTX and DEX differentially suppressed DTHA-induced paw swelling and inflammation. The effects of MTX and DEX coincided with differential regulation of levels of Th1, Th17, and memory T cells as well as B cells. Our results implicate Th1, Th17, and memory T cells, together with activated B cells, to be involved and required for DTHA-induced paw swelling and inflammation.

## Introduction

Rheumatoid arthritis (RA) is an inflammatory autoimmune disease elicited by complex interactions between genetic and environmental factors, leading to chronic life-long inflammation of synovial joints (1). Over time this may lead to progressive and severe joint destruction and deformity (2). The hallmark of RA-associated inflammation is the recruitment of a variety of immune cells, including neutrophils, monocytes/macrophages, B lymphocytes (B cells), and CD4+ and CD8+ T lymphocytes (T cells) to the synovial compartment, where pro-inflammatory cytokines and chemokines are produced, together contributing to the pathogenesis of RA (3–5). Although the etiology of the disease remains elusive, aberrant pro-inflammatory CD4+ T cell activity plays a central role in the initiation and perpetuation of RA (6, 7). The two most pronounced CD4+ T cell subsets involved in RA are thought to be CD4+ T helper 1 (Th1) cells and T helper 17 (Th17) cells (8–14). Moreover, memory CD4+ T cells have been found to be enriched in inflamed synovium, assisting B cell activity and Ig production (15, 16). For treating the RA, methotrexate (MTX) is the most versatile drug used for preventing joint damage and glucocorticoids (GCs) for suppressing inflammation. The combination of these two compounds is most frequently used to reduce RA progression (17–20).

In order to understand etiology and pathology of RA, and to explore potential novel therapeutic drugs and strategies, several animal models, which can mimic and resemble that of human RA, have been developed. These include collagen-induced arthritis (CIA), antigen-induced arthritis (AIA), collagen antibody-induced arthritis (CAIA), and delayed-type hypersensitivity arthritis (DTHA) mouse models (21–24). These models all differ in their mode of induction and strain susceptibility to RA development. The DTHA model was initially developed by Tanaka and coworkers and developed further by Atkinson, showing it to mimic several histopathological features of human RA (24, 25). The DTHA model was established in both BALB/c and C57BL/6 mice strains, and exhibits high incidence rate, low variation, and synchronized onset of disease. These characteristics make DTHA model a promising translational murine model with high pharmacological values.

DTHA develops in two phases, the immunization and challenging phase. In the immunization phase, mBSA is injected subcutaneously (s.c.) and mBSA-specific T cells are generated. In the challenge phase, recall responses of the mBSA-specific T cells are induced by injection of mBSA in one of the footpads, contributing to the release of pro-inflammatory cytokines which trigger the recruitment of inflammatory cells such as neutrophils and macrophages at the site of inflammation (26–30). This activity initiates a process that leads to synovial hyperplasia, pannus formation, and destruction of bone and cartilage during disease development. Additionally, an i.p. injection of anti-CII is given to mice between these two mBSA injections to enhance immune response. In the DTHA mouse model, inflammation generally reaches a maximum at 24–48 h after the second mBSA injection, and induction of inflammation and paw swelling relies on CD4+ T cell activity, as antibody-depletion of CD4+ T cells prevents DTHA development (24, 25). The anti-inflammatory subset of CD4+ T cells, regulatory T cell (Treg) has significant influence on DTHA-induced paw swelling since depletion of Treg can exacerbate DHTA severity (31). However, other pro-inflammatory CD4+ T cell subsets, for example, activated, Th1, and memory CD4+ T cells, have not been explored in this model.

In the DTHA model, even though some anti-inflammatory agents such as neutralizing antibodies to TNF-α and IL-17 have been shown effective in reducing inflammation, MTX, has to our knowledge not been explored in the DTHA model (24, 31). MTX interferes with folate-related metabolisms including *de novo* purine and pyrimidine synthesis and promotes production of the anti-inflammatory metabolite adenosine. By this, MTX treatment can inhibit T cell proliferation, induce T cell apoptosis, suppress neutrophil migration, and influence cytokine production (18, 32–39). Moreover, the effect of the synthetic glucocorticoid dexamethasone (DEX) on paw swelling has been shown. However, the effects of DEX on pro-inflammatory cells associated with DTHA have not been investigated (24). Despite this, DEX has been shown to attenuate T cell function either by directly affecting TCR-induced activation or indirectly enhancing PD-1 expression (40, 41).

Here we examined the identity of immune cells involved and compared the effects of MTX and DEX on DTHA-induced inflammation and paw swelling in C57BL/6 mice. Our results demonstrate that Th1, Th17, and memory T cells interplay with B cells in promoting DTHA-induced inflammation and paw swelling.

## Materials and Methods

### Cell Culture

Mouse CD4+ T cells were obtained from splenocytes using Dynabeads untouched CD4+ T cell isolation kit (Thermo Fisher). Mouse purified CD4+ T cells were cultured in RPMI-1640 medium supplemented with 10% heat-inactivated fetal bovine serum, 2 mM L-glutamine, 10 mM HEPES buffer solution, 1 mM sodium pyruvate, 100 µM MEM non-essential amino acid solution, 50 µM β-mercaptoethanol, 100 U/ml penicillin, 100 ug/ml streptomycin, and 30 U/ml human recombinant IL-2 (all the materials for cell culture are from Sigma-Aldrich). These purified CD4+ T cells were treated with incremental concentrations of MTX (10–1,000 nM; Sigma-Aldrich) overnight followed by stimulation with anti-CD3/CD28 beads (bead:cell = 1:1; Thermo Fisher). Post 3 days incubation, cells were collected for cell activation assay by flow cytometry (see *Flow Cytometry*).

### Mice and Ethics Statement

Female C57BL/6 mice (8–10 weeks old) were purchased from Charles River and housed in the local animal facility of University of Oslo. Mice were kept under 12-h light/dark cycle, with standard rodent chow *ad libitum* and drinking water. All animal experiment procedures were approved and registered at the National Animal Research Authority (FOTS ID: 5714, 19551, and 13903).

### Induction of DTHA

**Figure 1:**
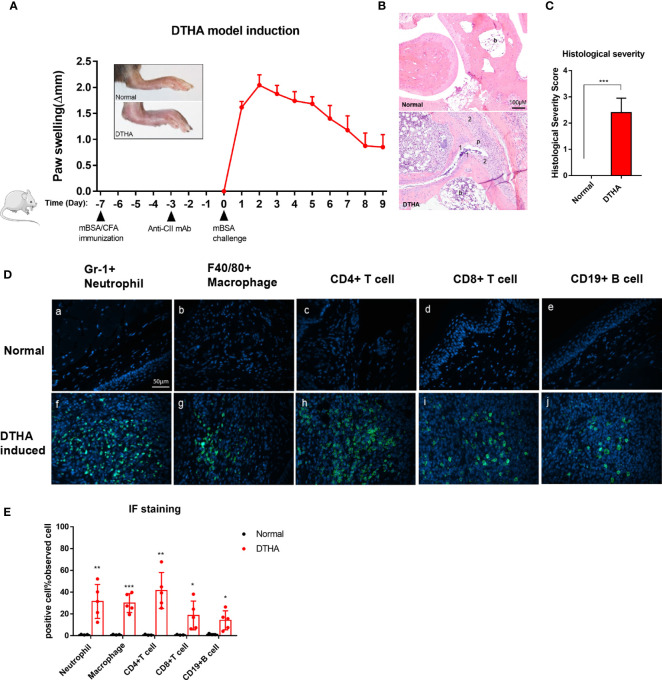
Swelling, inflammatory severity score, and immune cell infiltration of paws induced by DTHA in mice. **(A)** The timeline of DTHA induction in mice by immunization with 100 µg mBSA on day minus 7 (day −7), injection of anti-CII (50 mg/kg) on day −3 and 200 µg mBSA challenge at day 0. Paw swelling was followed between day 0 and 9 post the final 200 ug mBSA challenge. **(B, C)** Representative H&E stained sections (3.5 µM) of the paws **(B)** and histological severity score **(C)** from DTHA-induced and normal mice on day 9. Original magnification is 200×; scale bar, 100 μm; b: bone marrow; p: pannus; 1: destruction of joint cartilage; 2: Inflammation infiltration. Histology scores were calculated by the evaluation scale (see *Materials and Methods*). **(D)** Representative images of immunofluorescence staining for neutrophils (Gr-1+), macrophages (F4/80+), CD4+ T cells (CD4+), CD8+ T cells (CD8+), B cells (CD19+) infiltrating paws from normal (a–e) and DTHA-induced mice (f–j) on day 9. Original magnification, × 400; scale bar, 50 μm. Hoechst 33342 (blue colour) was used for nuclear counterstaining, while corresponding surface marker was shown in green colour. **(E)** Quantification of Gr-1+ neutrophils, F4/80+ macrophages, CD4+ T cells, CD8+ T cells, and CD19+ B cells. Values are expressed as the mean ± SD. All data are representative of two independent experiments with n = 3 in the normal group and n = 5 in DTHA group per experiment. Statistical significance was calculated by Student’s *t*-test to measure significant difference between normal group and DTHA group. (*p < 0.05, **p < 0.01, ***p < 0.001). The mouse image is from smart.servier.com.

Induction of DTHA is outlined in **Figure 1A**. In brief, mice were immunized intradermally (i.d.) at both sides of the lower back with 50 µg mBSA emulsified in 50 µl CFA (Hooke Laboratories) in each side on day −7 and injected i.p. with anti-CII cocktail (50 mg/kg, MD Bioscience) in 100 µl PBS on day −3. Seven days after initial immunization, mice were challenged with mBSA (5 mg/ml) in 40 µl PBS (Hooke Laboratories) subcutaneously in the left footpad and with vehicle (PBS, Sigma-Aldrich) in the right footpad, which serves as an intra-animal control (day 0). After 3–7 days, acute inflammation and severe arthritis rapidly developed in the mBSA-challenged paw. Mice were terminated on 1 day or 9 days after the mBSA challenge.

### MTX and DEX Administration

To determine the efficacy of MTX, mice were given MTX (0.75, 1.5, 3, 6 mg/kg i.p., Sigma-Aldrich) dissolved in 0.5% DMSO-containing PBS or the same volume of 0.5% DMSO-containing PBS (negative control) every other day from 2 weeks before mBSA immunization. In the DEX treatment group, mice were injected (i.p.) daily with 1 mg/kg DEX (Sigma-Aldrich) from the DTHA onset (day 0) to the end of study.

### Measurement of Paw Swelling and Histopathology

Clinical signs of arthritis in mice were recorded every day by measurement of paw thickness using a dial thickness gauge (Mitutoyo) after challenge with mBSA. The severity of hind paw swelling was evaluated as the increase in thickness of left inflamed paw (Δthickness = left inflamed paw thickness – right control paw thickness). On the termination day, mBSA-challenged left paws were dissected, fixed in 4% paraformaldehyde (VWR), decalcified in 7% EDTA solution for 2 weeks, and then embedded in paraffin blocks. For general assessment of histopathology, the paraffin-embedded tissues were sectioned (3.5 µm) and stained by hematoxylin and eosin (H&E). The histopathologic score was evaluated by a 1–4 scale: 1, hyperplasia of the synovial membrane and presence of inflammation infiltration; 2, pannus and cartilage erosion; 3, major erosion of cartilage and subchondral bone; and 4, loss of joint integrity and ankyloses (42). For each sample, three joint areas were selected, and the average histopathologic score was used for calculating histological severity score.

### Myeloperoxidase Activity Examination by IVIS Imaging

To determine myeloperoxidase (MPO) activity, DTHA mice were injected i.p. with 150 µl/mouse XenoLight RediJect Inflammation probe (Perkin Elmer), a chemiluminescent substrate of MPO, 1 day after mBSA challenge. Luminescence images were captured by SpectrumCT *In Vivo* Imaging System (IVIS; Perkin Elmer) 10 min after injection of the probe. The images were acquired with the following parameters (f/stop = 1; exposure time = 180 s; binning factor = 8). The results were expressed as the intensity of radiance (photons/s/cm^2^/sr) using Living Image software (version 4.2; Perkin Elmer).

### Assessment of Immune Cell Infiltration in Arthritic Paws by Immunohistochemistry

To detect infiltrated immune cells, paw sections (3.5 µm) were fixed with acetone and denatured with 100, 95, and 75% ethanol for 15 s separately. For antigen retrieval, sections were heated in Tris-EDTA retrieval buffer (10 mM Tris Base, 1 mM EDTA solution, 0.05% Tween 20, pH 9.0) in a water bath at 98°C for 20 min. After cooling down at RT for 30 min, sections were incubated with antibodies against F4/80 (clone CI:A3-1; Abcam), GR-1 (clone RB6-8C5; R&D), CD4 (clone 4SM95; Thermo Fisher), CD8 (clone 4SM15; Thermo Fisher), and CD19 (clone 6OMP31; Thermo Fisher) at 4°C overnight for detecting macrophages, neutrophils, CD4+ T cells, CD8+ T cells, and B cells respectively. Next, sections were washed with PBS and incubated with anti-Rat IgG (Alexa Fluor 488 conjugated; Thermo Fisher) secondary antibody. Cell nucleus was counterstained with Hoechst 33342 (Thermo Fisher), and sections were visualized using a fluorescence microscope (Olympus BX61). Quantification of fluorescence intensity was performed by count function in the software of CellSens dimension (Olympus). Briefly, five different regions in arthritis paw from DTHA mice and normal paw from untreated mice with strongest fluorescence intensity were selected for each sample. Then the average value of fluorescence intensity was calculated from these five regions and used for the next statistical analysis.

### Flow Cytometry

In order to detect pro-inflammatory cells in DTHA, single-cell suspensions were prepared from draining inguinal lymph node (iLN) and spleen of mice in different groups on the termination day (day 1 or/and day 9). To quantify CD4+ T cells (CD4+), activated CD4+ T cells (CD4+CD69+CD25+), central memory CD4+ T cells (CD4+CD62L+CD44+), effector memory CD4+ T cells (CD4+CD62L−CD44+), Th1 (CD4+ CD183+ CD194−CD196−), Th17 (CD4+CD183− CD194+CD196+), and B cells (B220+CD4−), corresponding antibodies were used for incubation (11, 43, 44). Please find the information of all antibodies for flow cytometry in **Supplementary Table 1**. Post incubation with antibodies, samples were washed and analyzed by FACS Canto II flow cytometer (BD Biosciences). Flow data analyses were done in FlowJo V10 (BD Biosciences).

### ELISA Assay for Antibody Determination

Nine days post mBSA challenge, blood samples were collected from DTHA mice treated with PBS or MTX and then centrifuged for 15 min at 2,000 g for plasma preparation. Murine total IgG antibody levels in the plasma were examined by enzyme-linked immunosorbent assays (ELISAs) as per the instructions (Thermo Fisher). Briefly, ELISA plates were coated with purified anti-mouse IgG monoclonal antibody overnight at 4°C, washed three times with wash buffer (PBS containing 0.05% Tween-20 and 1% BSA), and finally blocked with PBS containing 1% BSA overnight at 4°C. Next day, serial dilutions of plasma (diluted at 1:625, 1:2,500, and 1:10000) were added. The plates were detected using horseradish peroxidase-conjugated anti-mouse IgG, followed by TMB substrate. Finally, stop buffer was added, and optical density was measured at 450 nm. Antibody concentration for each sample was calculated by using standard curve.

### Statistical Analysis

All significance values between two groups were determined by the unpaired Student’s t-test. Comparison between more than two groups was analyzed with ANOVA with Tukey multiple comparison test. The correlation between two variables was calculated by Pearson correlation test. Data are presented as the mean ± the standard deviation, and all the statistical analyses were performed by GraphPad Prism 9 (GraphPad Software). P<0.05 was considered a statistically significant difference, and levels of significance were assigned as *P ≤ 0.05, **P ≤ 0.01, ***P ≤ 0.001, and ****P ≤ 0.0001.

## Results

### Establishing the DTHA Mouse Model

The DTHA model in mice was established as described in the *Materials and Methods* and by others and according to the timeline depicted in **Figure 1A** (24, 25). In line with the observations of others, paw swelling was detected and measured 12–24 h post the mBSA challenge denoted day 1 (see *Materials and Methods* and **Figure 1A**). Histopathological examination of tissue sections by H&E staining 9 days post mBSA challenge revealed synovial inflammatory infiltration, pannus formation, and bone/cartilage erosion in DTHA-induced paw (**Figures 1B, C**). In RA, inflammation is associated with the recruitment of a variety of immune cells. Based on this we next qualitatively and quantitatively investigated the identity and levels of infiltrated immune cell composition by immunofluorescence in the paws of DTHA compared to normal unaffected mice, respectively. We observed a significant increase in the levels of GR1+ neutrophils, F4/80+ macrophages, CD4+ T cells, CD8+ T cells, and CD19+ B cells in the paws of DTHA-affected mice compared to normal mice (**Figures 1D, E**).

### Levels of Activated CD4+ T Cells in Lymph Nodes and Spleen of DTHA Mice Are Elevated

**Figure 2:**
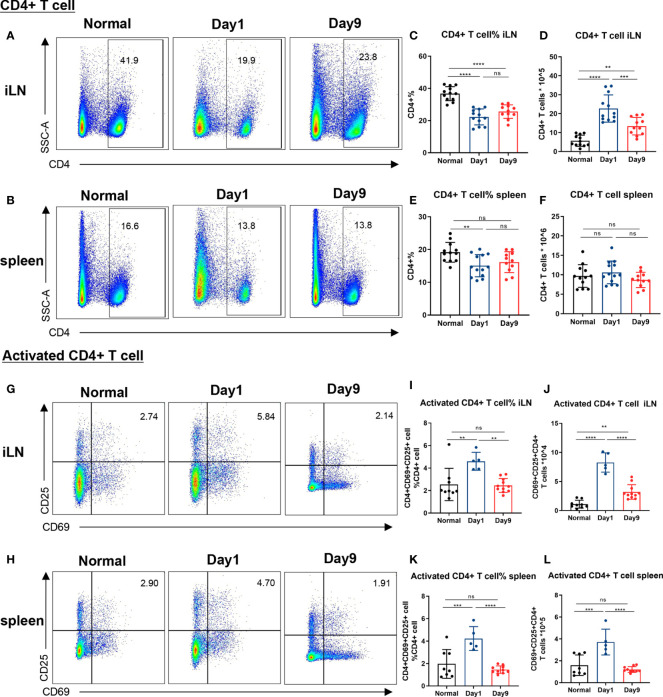
Levels of CD4+ and activated CD4+ T cells in lymph nodes and spleen of DTHA mice. Relative and absolute levels of CD4+ and activated CD4+ T cells isolated from draining inguinal lymph node (iLN) and spleen of normal mice and DTHA-induced mice on day 1 and day 9 post the mBSA challenge. **(A, B)** Representative flow cytometry dot plot of CD4+ T cell from iLN **(A)** and spleen **(B)**. **(C–F)** Quantification of relative and absolute number of CD4+ T cells from iLN **(C, D)** and spleen **(E, F)**. **(G, H)** Representative levels of activated CD4+ T cells (CD4+CD69+CD25+) from iLN **(G)** and spleen **(H)**. **(I–L)** Relative number and absolute number of activated CD4+ T cells from iLN **(I, J)** and spleen **(K, L)** on day 1 and day 9. Data is presented as mean ± SD. All data pooled from two independent experiments (at least five mice per group in each experiment) with the exception of the data of activated CD4+ on day 1, which is from one experiment with five mice. Statistical significance was calculated by ANOVA with Tukey multiple comparison test (**p < 0.01, ***p < 0.001, ****p < 0.0001, ns: p > 0.05).

As mentioned, CD4+ T cells are indispensable for DTHA-induced inflammation and paw swelling (24, 25). Because of this, we measured the level of CD4+ T cells in the spleen and inguinal LN (iLN), the latter as representative paw-draining LN, on day 1 and 9 post DTHA onset. This demonstrated that the proportions of CD4+ T cells in both iLN and spleen were reduced significantly, despite that the absolute numbers increased in iLN and remained the same in spleen (**Figures 2A–F**). In accordance with this result and the fact that the mBSA challenge was expected to induce extensive cell division, we measured the level of activated CD4+ T cells by staining for the CD25 and CD69 cell surface markers (45). This demonstrated that the absolute and relative number of activated CD4+ T cells (CD4+ CD25+CD69+) on day 1 after the mBSA challenge were significantly elevated and declined over the time span investigated (days 1 through 9, **Figures 2G–L**).

### Differential Induction of Th1 and Th17 Cells in DTHA Mice

**Figure 3:**
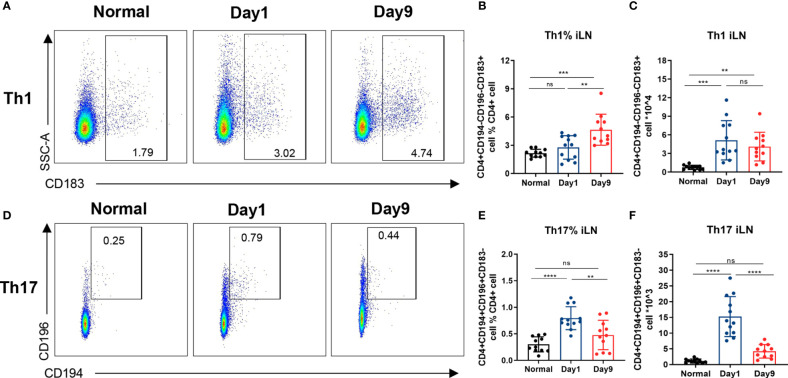
Differential induction of Th1 and Th17 cells in DTHA mice. Relative and absolute levels of Th1 and Th17 cells in draining iLN of normal and DTHA mice on day 1 and day 9 post mBSA challenge analyzed by flow cytometry. **(A)** Representative flow cytometry dot plot illustrating relative population of Th1 cells (CD4+ CD194−CD196−CD183+). **(B, C)** Fraction (%) **(B)** and absolute number **(C)** of Th1 cells on day 1 and day 9 post mBSA challenge. **(D)** Representative flow cytometry dot plot illustrating relative population of Th17 cells (CD4+CD194+CD196+CD183−). **(E, F)** Fraction (%) **(E)** and absolute number **(F)** of Th17 cells on day 1 and day 9 post mBSA challenge. Data is presented as mean ± SD from two independent experiments with at least five mice in each group per experiment. Statistical significance was calculated by ANOVA with Tukey multiple comparison test (**p < 0.01, ***p < 0.001, ****p < 0.0001, ns: p > 0.05).

The Th1 and Th17 effector T cells are considered crucial players in the pathogenesis and development of RA (8–14). Flow cytometry analysis was used to quantify Th1 and Th17 cells isolated from iLN of mice challenged with mBSA on days 1 through 9. When comparing to normal mice, sustained elevation of both relative and absolute numbers of Th1 cells (CD4+CD183+CD194−CD196−) in iLN were observed in the DTHA-induced mice (**Figures 3A–C**). In contrast to Th1 cells, the relative and absolute number of Th17 cells (CD4+CD183−CD194+CD196+) in iLN exhibited a significant increase on day 1, which was transient and had decreased by day 9 (**Figures 3D–F**). We further explored the changes in splenic Th1 and Th17 cells and showed a significant increase in the relative number on day 9 (**Supplementary Figures 1A–D**). Together, these findings imply that levels of Th1 and Th17 cells are differentially upregulated during DHTA development.

### Central/Effector Memory CD4 + T Cells Are Increased in DTHA Mice

**Figure 4:**
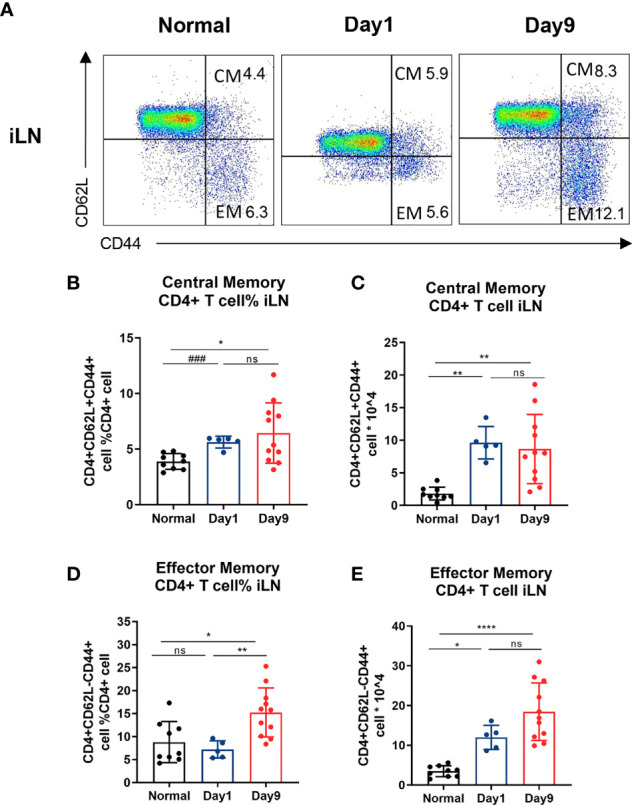
Central/effector memory CD4 + T cells are increased in DTHA mice. Cells were isolated from draining iLN of normal mice and DTHA-induced mice on day 1 and 9 post mBSA challenge and analyzed by flow cytometry. **(A)** Representative flow cytometry dot plot of central/effector memory CD4+ T cells from iLN from normal mice and DTHA-induced mice on day 1 and day 9 post mBSA challenge. **(B, C)** Relative number **(B)** and absolute number **(C)** of central memory CD4+ T cells (CD4+CD62L+CD44+) from iLN in given groups. **(D, E)** Relative number **(D)** and absolute number **(E)** of effector memory CD4+ T cells (CD4+CD62L−CD44+) from iLN are shown. Data is presented as mean ± SD. Data of naïve group and day 9 group are from two independent experiments with at least five mice per group per experiment. Data of day 1 group is from one experiment with five mice. Statistical significance was calculated by ANOVA with Tukey multiple comparison test (*p < 0.05, **p < 0.01, ****p < 0.0001, ns: p > 0.05) or Student’s *t*-test (^###^p<0.001). CM: central memory CD4+ T cell. EM: effector memory CD4+ T cell.

Levels of memory CD4+ T cells have been reported to be elevated in the inflamed synovium of RA patients and to be essential for the development of the DTH response (15, 16, 46). Memory CD4+ T cells may be subdivided into central and effector memory CD4+ T cells (47). Whereas, CD44 expression is enhanced on all memory T cells, differential expression of CD62L is commonly used to distinguish central memory from effector memory T cells (48, 49). When staining for CD44 and CD62L, we observed a significant long-term increase in both relative and absolute number of both central memory (CD4+CD62L+CD44+) and effector memory (CD4+ CD62L− CD44+) CD4+ T cells isolated from iLN in the DTHA mice (**Figures 4A–E**). This was identical to that of the splenic effector memory but not central memory CD4+ T cells (**Supplementary Figures 2A–E**). However, splenic central memory CD4+ T cells only showed a temporal increase over time.

### B Cell Levels Increase in Draining Lymph Node but Not Spleen in DTHA Mice

**Figure 5:**
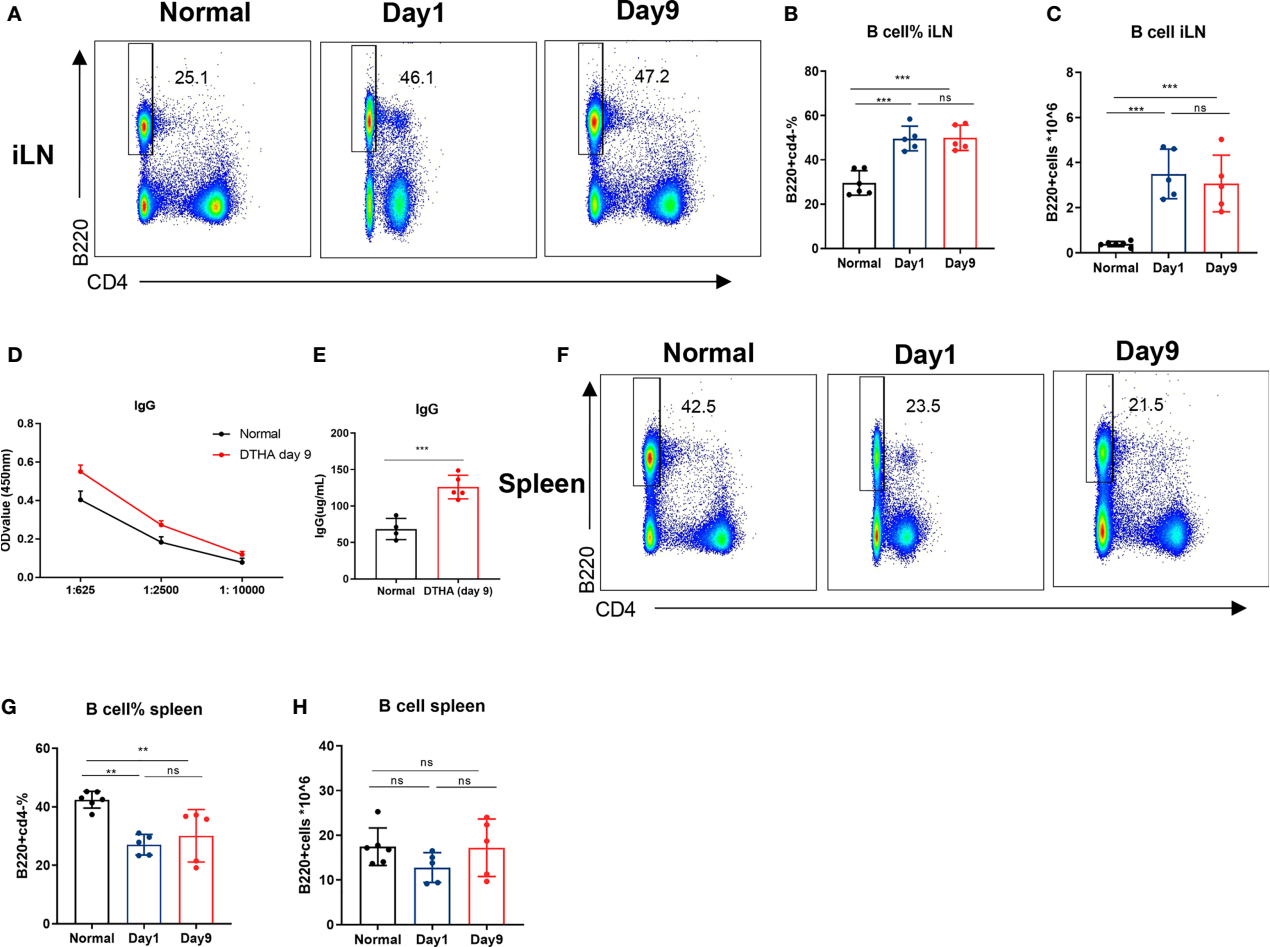
B cell levels increase in draining lymph node but not spleen in DHTA mice. Cells were prepared from the draining iLN and spleen of DTHA-induced and normal mice on day 1 and day 9 and analyzed by flow cytometry. **(A)** Representative flow cytometry dot plot of B cells (B220+CD4−) from iLN DTHA and DTHA mice on day 1 and day 9 post mBSA challenge. **(B, C)** The proportion **(B)** and absolute number **(C)** of B in iLN are shown. **(D)** On day 9, serial dilutions of plasma were analyzed for IgG antibody titers by ELISA from normal mice and DTHA-induced mice. **(E)** The levels of plasma IgG antibodies (prediluted 1:625) determined by ELISA in mice from each group on day 9. **(F)** Representative flow cytometry dot plot of B cells from spleen in normal mice and DTHA mice on day 1 and day 9 post mBSA challenge. **(G, H)** The proportion **(G)** and absolute number **(H)** of B in spleen are shown. All data are presented as mean ± SD and representative of two independent experiments with n = 3–4 in the normal mice group and n = 5 in DTHA group per experiment. Statistical significance was calculated by Student’s t-test for the comparison of two mouse groups (***p < 0.001). For the comparison of three groups, statistical significance was calculated by ANOVA with Tukey multiple comparison test (**p < 0.01, ***p < 0.001, ns: p > 0.05). ELISA, Enzyme-linked immunosorbent assay.

B cells are instrumental in the pathogenesis of RA as they are activated to produce rheumatoid factor, which, in addition to a number of other autoantibodies, is associated with RA initiation and pathogenesis (50). To investigate a potential role of B cells involvement in the pathogenesis of DTHA, we examined the relative and absolute numbers of B cells (B220+CD4−) in addition to levels of circulating IgG after the mBSA challenge on day 0. We observed a significant twofold increase in the proportion of B cells in iLN, which lasted throughout the study time (days 1 through 9) (**Figures 5A–C**). The rise in B cells coincided with elevated level of IgG in circulation (**Figures 5D, E**). It should be noted that the number of splenic B cells was decreased on day 1 and was restored back to initial levels on day 9 (**Figures 5F–H**).

### MTX Prevents DTHA-Associated Paw Swelling and Inflammation

**Figure 6:**
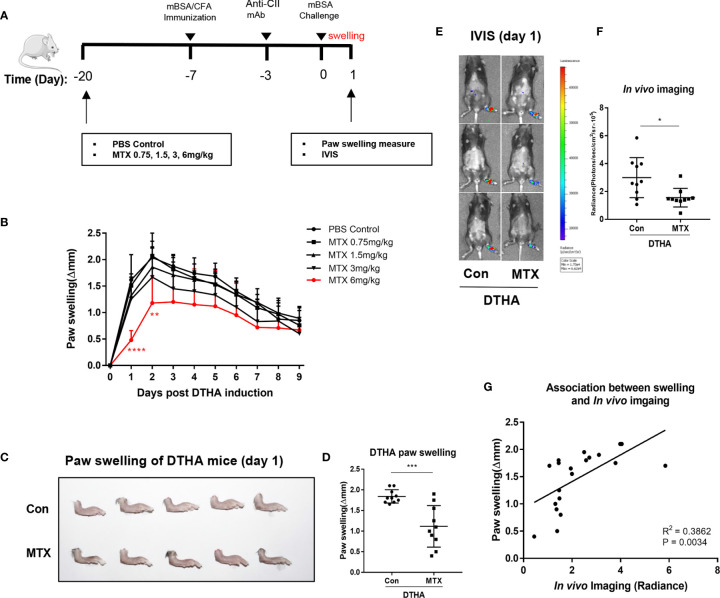
MTX prevents DTHA-associated paw swelling and inflammation. **(A)** MTX treatment regime during DTHA induction is illustrated. **(B)** Effect of different dose of MTX (0–6 mg/kg) on paw swelling were monitored from day 0 to day 9 post DTHA onset. **(C, D)** Representative appearance **(C)** and quantification **(D)** of paw swelling in DTHA mice treated with and without MTX (6 mg/kg). **(E)** Representative bioluminescent images indicting myeloperoxidase (MPO) activity of hind paws from DTHA mice treated with and without 6 mg MTX/kg on day 1. **(F)** Quantification of in *vivo* luminescence imaging for mice treated with and without MTX (6 mg/kg). **(G)** Linear regression analysis of paw swelling and in *vivo* luminescence imaging for MPO activity in DTHA mice treated with and without MTX (6 mg/kg). The data are representative of pooled data from two independent experiments with five mice in each group per experiment, with the exception of the data of 0.75 and 3 mg/kg MTX treatment, which are from one experiment with five mice. Statistical significance was calculated by Student’s t-test for the comparison of two mouse groups (*p < 0.05, **p < 0.01, ***p < 0.001, ****p < 0.0001). Stars under the curve in panel B represent significant difference in paw swelling between 6 mg MTX/kg treatment group and PBS-treated control group (**p < 0.01, ****p < 0.0001). The correlation between paw swelling and imaging was calculated by Pearson correlation test. Con, DTHA Control group. The mouse image is from smart.servier.com.

MTX has been demonstrated effective in attenuating inflammation in many experimental arthritis murine models, including the CIA and AIA models (51, 52). Despite this, the effects of MTX on inflammation and paw swelling have not been explored in the DTHA model. To determine the efficacy of MTX in the DTHA model, we initially monitored its effect on paw swelling. Because MTX is a slow-acting regimen, long-time administration before symptom onset was adopted from other inflammatory mice models (51, 53–55). We chose a protocol starting with treatment at day minus 20 of the mBSA challenge (**Figure 6A**). We tested incremental doses of MTX ranging from 0 to 6 mg MTX/kg (**Figure 6B**) and showed that 6 mg MTX/kg was required to significantly delay and reduce paw swelling. The most pronounced decline in paw swelling was apparent during the first 24 h after the mBSA challenge (day 0), where the average paw swelling (Δmm) decreased from 1.84 ± 0.16 to 1.11 ± 0.50 (**Figures 6C, D**). To determine the effect of MTX on the ongoing inflammatory activity caused by DTHA, we next assessed neutrophil infiltration by monitoring myeloperoxidase (MPO) activity. This showed that MPO activity was significantly reduced using 6 mg MTX/kg (**Figures 6E, F**). Finally, the level of luminescence intensity was positively correlated with paw swelling when administrating 6 mg MTX/kg on day minus 20 of the mBSA challenge (**Figure 6G**).

### Differential Effects of MTX and DEX on DTHA Mice

**Figure 7:**
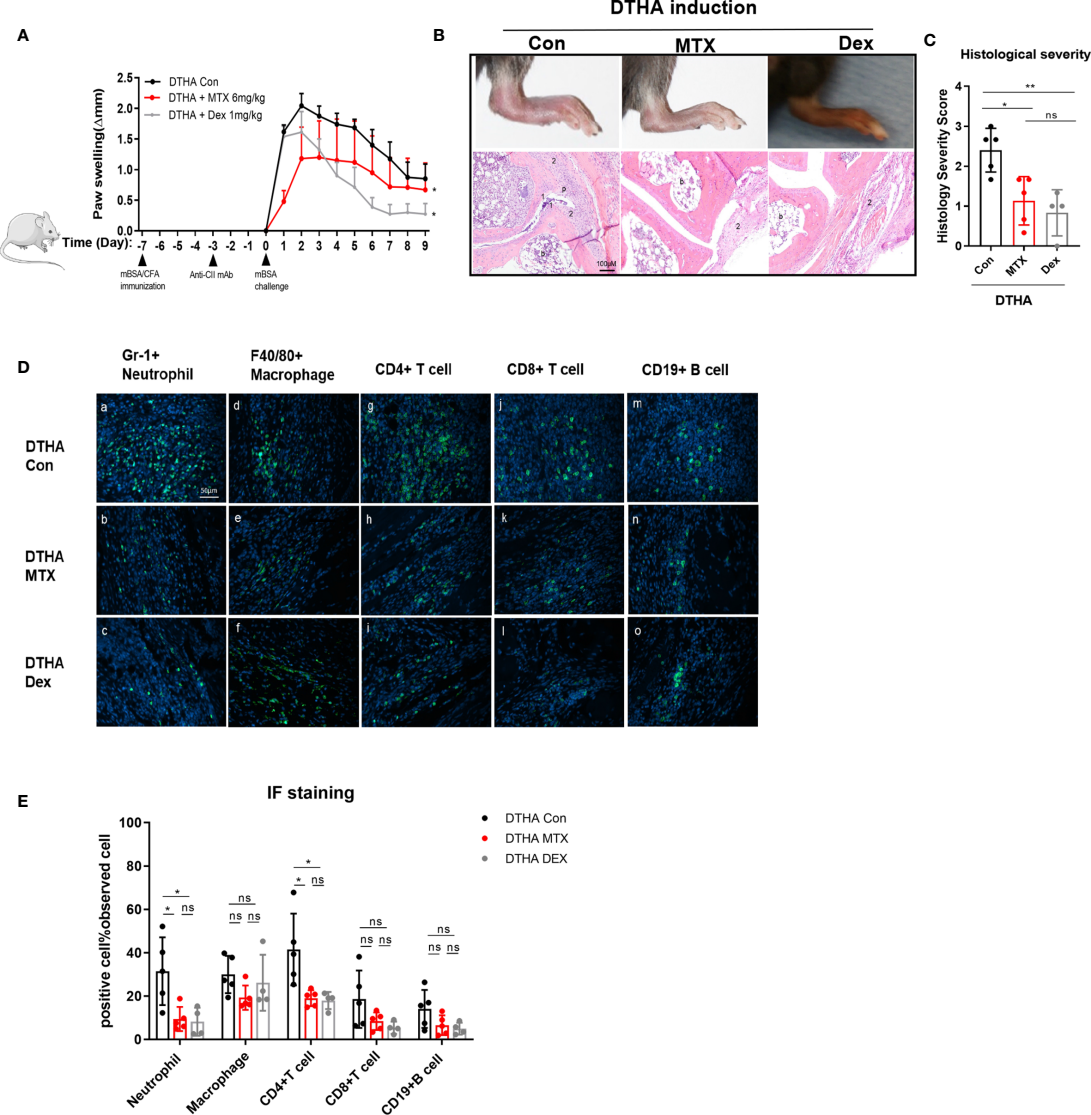
Differential effects of MTX and DEX on DTHA-induced paw swelling but not inflammation. **(A)** Dynamic effects of MTX (6 mg/kg) and DEX (1 mg/kg) on paw swelling from day 0 to day 9 after the mBSA challenge. Changes of paw swelling (Δmm) were used to determine effects of MTX and DEX. (*) significant difference in paw swelling between DTHA mice treated with or without MTX or DEX. Data are representative of two independent experiments (n = 4–6 mice in each group). **(B)** Representative H&E-stained sections (3.5 µM) of the paws were analyzed from one experiment from DTHA mice treated without (n = 5 mice) or with MTX (n = 5 mice) or DEX (n = 4 mice) on day 9. Original magnification is ×200; scale bar, 100 μm; b: bone marrow; p: pannus; 1: destruction of joint cartilage; 2: inflammation infiltration. **(C)** Histology scores were calculated by the evaluation scale (see *Materials and Methods*). **(D)** Representative images of immunofluorescence staining on day 9 for neutrophils (Gr-1+) (a–c), macrophages (F4/80+) (d–f), CD4+ T cells (CD4+) (g–i), CD8+ T cells (CD8+) (j–l), B cells (CD19+) (m–o) of paws from one experiment from DTHA mice treated without (n = 5 mice) or with MTX (n = 5 mice) or DEX (n = 4 mice). Original magnification, ×400; scale bar, 50 μm. Hoechst 33342 (blue color) was used for nuclear counterstaining, while corresponding surface marker was shown in green color. **(E)** Quantification of Gr-1+ neutrophils, F4/80+ macrophages, CD4+ T cells, CD8+ T cells, and CD19+ B cells. Values are expressed as the mean ± SD. Statistical significance in panel A was calculated by Student’s *t*-test (*p < 0.05) to compare the difference between DTHA control mice and DTHA mice treated with MTX or DEX. Statistical significance in panels C and E was calculated by ANOVA with Tukey multiple comparison test (*p < 0.05, **p < 0.01, ns: p > 0.05). Con, DTHA Control group. The mouse image is from smart.servier.com.

DEX is a potent anti-inflammatory drug used in RA treatment (19, 20, 40, 41). Due to this, we also tested the effects of DEX in the DTHA model and compared the effects to MTX. In contrast to MTX, which had a marked effect on the early stage of DTHA development (between day 0 and 3), DEX exerted a more delayed but sustained effect on paw swelling from days 1 through 9 post the mBSA challenge (**Figure 7A**). It should however be noted that DEX treatment, in contrast to MTX, negatively affected the weight of the mice over time (**Supplementary Figures 3A–C**). This was apparent, despite that the MTX and DEX were administrated from day minus 20 and day 0 or day minus 8, respectively. This discrepancy suggested differential modes of action and prompted us to assess their effects by examining histological severity and inflamed paw cell infiltration. We found that both MTX and DEX ameliorated DTHA-induced histological severity (**Figures 7B, C**). Moreover, both MTX and DEX prevented paw immune cell infiltration, which was associated with reduced levels of neutrophils and CD4+ T cells, whereas macrophages, CD8+ T cells, and CD19+ B cells were not altered significantly (**Figures 7D, E**).

**Figure 8:**
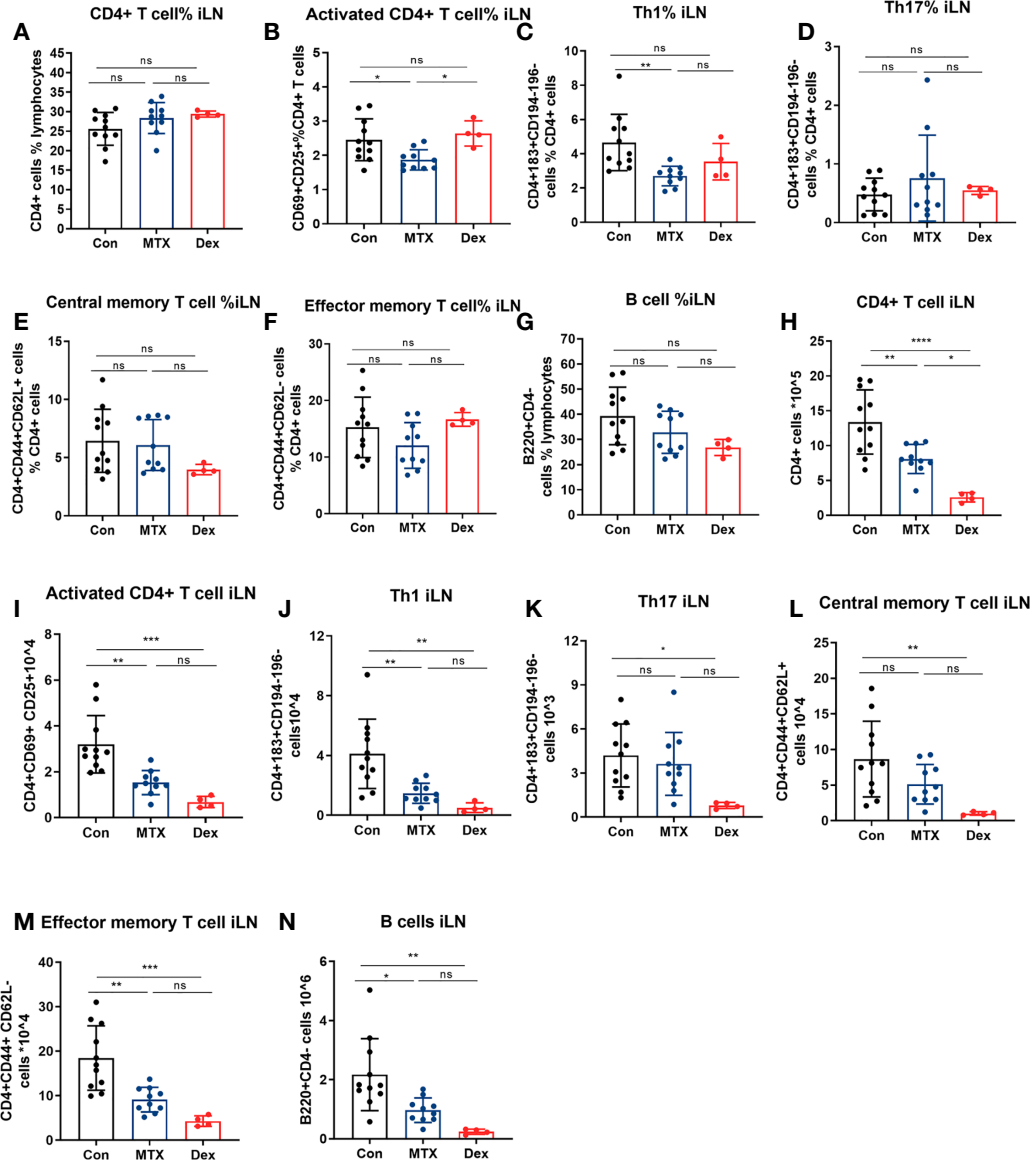
Differential effects of MTX and DEX on levels of DTHA-induced immune cells. Cell suspensions were prepared from the draining iLN of DTHA mice treated without or with MTX or DEX on day 9. CD4+ T cell, activated CD4+ T cell, Th1, Th17, central/effector memory T cell, and B cell were analyzed by flow cytometry. The proportion and absolute number of CD4+ T cell **(A, H)**, activated CD4+ T cell **(B, I)**, Th1 **(C, J)**, Th17 **(D, K)**, central memory CD4+ T cell **(E, L)**, effector memory CD4+ T cell **(F, M)** and B cell **(G, N)** in iLN from mice in given groups are shown.. All data are presented as mean ± SD. The DEX group data is from one experiment. All other data are pooled from two independent experiments with three to six mice in each group per experiment. Statistical significance was calculated by ANOVA with Tukey multiple comparison test (*p < 0.05, **p < 0.01, ***p < 0.001, ****p < 0.0001, ns: p > 0.05). Con, DTHA Control group.

To further examine and compare the effects of MTX and DEX, we monitored the relative numbers of immune cells in iLN on day 9. We found that MTX but not DEX treatment suppressed accumulation of the relative number of activated CD4+ T cells and Th1 cells in the iLN (**Figures 8A–G**). We also noted that the DEX treatment led to notable decrease in the absolute number of CD4+ T cells subsets residing in the iLN (**Figures 8H–N**). This coincided with a marked size reduction of the iLN with decreased cell number after the DEX treatment (**Supplementary Figure 4A**). Such shrinkage was also found for the spleen and coincided with reduced total numbers of splenocytes after the DEX treatment (**Supplementary Figures 5A–G, 4B**). By contrast, MTX did not have any effect on the absolute number of splenic immune cells in general. In fact, the MTX treatment was only associated with a reduction of the relative number of splenic Th1 cells. Together with its effect on iLN Th17 cells, this may indicate that the inhibitory effect of MTX on Th1 occurs through altered differentiation (**Supplementary Figures 5H–N**). In addition to T cells, the effects of MTX and DEX on splenic but not iLN B cell levels were differentiated (**Figures 8M, N** and **Supplementary Figures 5G, N**). To this end, the relative number of splenic B cells were reduced by MTX but not DEX, while the absolute number decreased with DEX (**Supplementary Figures 5G, N**). Finally, we found that 6 mg/kg MTX administration from day minus 20 in contrast to 1 mg/kg DEX exerted no effect on mice weight (**Supplementary Figures 3A–C**). Taken together, we observed that MTX suppressed immune cell numbers in a subset-specific manner, while DEX affected all immune cells investigated.

## Discussion

Well-defined preclinical animal models are required to better understand the mechanism of disease pathologies and how to target autoimmunity arthritis (17–20, 42, 43). The DTHA mouse model has recently been established and provides a promising tool to study RA initiation, development, and targeting (20). In this study, paw swelling, inflammation, and levels of inflammatory immune cells associated with DTHA were studied in the presence and absence of the two RA drugs, MTX and DEX.

Consistent with previous studies, DTHA mice exhibited sustained paw swelling and histological manifestations such as synovitis, synovial hyperplasia, and pannus formation, together with infiltration of inflammatory cells (24). The latter included infiltration of neutrophils, macrophages, CD4+ or CD8+ T cells, and B cells at the site of inflammation. These clinical signs are reminiscent of human RA and of the experimental RA mouse models, CIA, AIA, and CAIA model (56–58). It has been shown that paw swelling in the DTHA model depends on the presence and activity of CD4+ T cells and can be augmented by depletion of regulatory T cells (Tregs) (20, 44). Together these findings suggest the mechanism underlying DTHA inflammation requires the presence of CD4+ T cells. We could confirm this in that elevated levels of activated CD4+ T cells were detected in both iLN and spleen on day 1 post the mBSA challenge. Interestingly, the total CD4+ T cells proportion in iLN and spleen of the DTHA mice was significantly reduced rather than increased as compared with normal non-affected mice, although with elevated absolute numbers in iLN. We speculate that this may be a sign of migration of CD4+ T cells to inflamed sites or generation of more proliferated non-CD4+ T cells in these peripheral immune organs.

To investigate this further, the level of CD4+ T cell effector subsets, including Th1 and Th17 cells, were studied. Th17 cells are considered crucial for the development of both RA in human and for the pathophysiological development of CIA in mice (59, 60). We found that Th17 cells were significantly increased in iLN in the DTHA mice. This may be indicative of Th17 cells involvement in DTHA development and is in line with previous studies where neutralizing Th17 cell-specific cytokine IL17 counteracted DTHA-induced inflammation (31). To further study the involvement of CD4+ T cell subsets during DTHA development, levels of Th1 cells were examined. Th1 cells are considered pivotal effector CD4+ T cells involved in RA development in humans and in the DTH response in mice (6). In contrast to Th17 cells, levels of Th1 cells increased in the iLN on day 1, and a significantly twofold elevation was observed on day 9 post the mBSA challenge. Elevated Th1 levels were also observed in the spleen on day 9. The fact that the Th17 cell population was only transiently elevated may suggest that these cells are involved in the early stage of DTHA initiation and development. By contrast, Th1 levels, which remained high throughout the time of observation, may be involved in the regulation of sustained paw swelling. Hence, the distinct dynamics of the Th1 and Th17 cell levels throughout the time of observation (days 1 through day 9) suggest differential roles for these T cells subsets in the initiation and development of DTHA. Moreover, our observation of early elevation of Th17 cells followed by a decline that coincided with a sustained upregulation of Th1 may implicate transdifferentiation of Th17 into Th1 during the first 9 days of DTHA. This suggestion finds support in that Th17 cells display a high degree of context-dependent plasticity and may transdifferentiate into Th1 under certain conditions of autoimmunity including RA (61–64). To what extent this is the case during DTHA development needs further investigation. However, and despite these differences, we suggest that both Th1 cells and Th17 cells are involved and important for DTHA-associated inflammation from day 1 of the mBSA challenge.

Elevated levels of memory CD4+ T cells are expressed in the synovial fluid of affected joints in RA patients and are thus suggested to play a role in the pathogenesis of RA (15). We observed a marked and persistent increase in memory T cells in the iLN of DTHA mice, defined as effector and central memory CD4+ T cells, respectively. In the DTH-induced models, memory CD4+ T cells are shown to emerge from antigen-specific T cells within 5–7 days after an initial exposure to the antigen (22, 23, 26, 49). In the present study, the DTHA mice were immunized with mBSA 7 days prior to the mBSA challenge. Consistent with the reports of others, memory CD4+ T cells in the present experimental setup were successfully observed from 7 days post mBSA immunizing event, hence indicating that memory T cells are likely to be involved in the inflammatory response to the mBSA challenge. Recently it has been hypothesized that memory CD4+ T cells are generated from early commitment of Th1 and Th17 cells (65–67). Moreover, it has been reported that peripheral and synovium memory CD4+ T cells express Th1 phenotypic CD183 and IFN-γ, respectively (68, 69). To this end, it should be noted that levels of central/effector memory CD4+ T cells coincided with Th1 cells, supporting a potential association of memory and Th1 cells in the DTHA mouse model. Finally, as mentioned, B cells have been suggested not to be responsible for DTHA development since B cell depletion does not affect DTHA-associated paw swelling (24, 25). However, the B cell-produced autoantibodies have been reported to be associated with increased bone erosion in RA (70, 71). Based on this, we speculated that B cells may play a role in DTHA through promoting bone destruction–related manifestation, which was marked in the DTHA mice, rather than intensifying paw swelling directly.

To substantiate further the role of the pro-inflammatory cells involved in DTHA, we also investigated the effect of the anti-rheumatoid drug MTX and the anti-inflammatory drug DEX on paw swelling and immune cell infiltration in the swollen paw. As the effects of MTX have not been studied in the DTHA model, we tested its effect by titrating the dose and found that 6 mg MTX/kg was required for successful prevention of DTHA-associated paw swelling and inflammation. We noted that 6 mg MTX/kg is markedly higher compared to the dose of MTX required to dampen inflammation in the CIA and the AIA mice models, which is between 0.75 and 3 mg MTX/kg (51, 52). This may imply that DTHA inflammation is less sensitive to MTX compared to inflammation in the CIA and AIA models. We speculate that this may in part be caused by the anti-CII at day minus 3. Anti-CII has been reported by others to render the inflammatory response less sensitive to MTX treatment (72). Moreover, consistent with a previous study, administration of a low dose (1 mg/kg) of DEX suppressed DTHA-associated paw swelling in a more potent and sustained fashion (24). Specifically, DEX was effective immediately after administration at day 0, whereas MTX had to be administrated at day −20 upon the mBSA challenge. These observations strongly point to differential modes of action of DEX and MTX, which was supported in that the two drugs displayed differential effect on the levels of pro-inflammatory cell in the iLN and spleen. MTX reduced the proportion of activated CD4+ T cells in both DTHA mice and *in vitro* (**Supplementary Figure 6**). Additionally, MTX diminished DTHA-induced increased level of Th1 cells, which is consistent with previous studies showing that MTX can effectively suppress both Th1 cell population and its cytokine production, such as IFN-γ (73–75). This was in contrast to DEX, which failed to affect any of CD4+ T cell subsets proportion in iLN. Instead, DEX treatment markedly reduced cellularity of both iLN and spleen and thus reduced the absolute number of CD4+ T cell subsets in these immune tissues. Furthermore, DEX but not MTX treatment was associated with weight loss. Based on this, we conclude that DEX exerts a broader inhibitory effect on pro-inflammatory cells compared to MTX.

Results from this study document the dynamics of Th1, Th17, and memory CD4+ T cells, as well as activated B cell-induction during DTHA-induced paw swelling. Furthermore, the two widely prescribed RA drugs, MTX and DEX, revealed differential effects on paw swelling and levels of pro-inflammatory cells, further confirming a vital role for Th1, Th17, and memory T cells, as well as B cells, in contributing to DTHA in mice.

## Data Availability Statement

The original contributions presented in the study are included in the article/**Supplementary Material**. Further inquiries can be directed to the corresponding author.

## Ethics Statement

The animal study was reviewed and approved by National Animal Research Authority (Norway).

## Author Contributions

GL designed, performed, analyzed the experiments and contributed to write the manuscript. SK designed, assisted with the experiments, and contributed to writing the manuscript. SG, KM, and GM helped with experiments that involved mice and revised the manuscript. PK assisted with the experiments. FG designed the experiments and revised the manuscript. GH revised the manuscript. BS led the project, designed, supervised the experiments and their analyses, and contributed to write the manuscript. All authors contributed to the article and approved the submitted version.

## Funding

This work was supported by the UiO-MED and UiO-IMB, Grants to GL, SG, and BSS, The Norwegian Research Council, grant #ES612356, and Throne Holst Foundation, grant # BSS2019/2021. Principal funding recipient is BSS.

## Conflict of Interest

The authors declare that the research was conducted in the absence of any commercial or financial relationships that could be construed as a potential conflict of interest.

## Publisher’s Note

All claims expressed in this article are solely those of the authors and do not necessarily represent those of their affiliated organizations, or those of the publisher, the editors and the reviewers. Any product that may be evaluated in this article, or claim that may be made by its manufacturer, is not guaranteed or endorsed by the publisher.

## Abbreviations

DTHA, delayed-type hypersensitivity arthritis; H&E, hematoxylin and eosin; IF, immunofluorescence; Th, helper T cell type; IL, interleukin; PBMC, peripheral blood mononuclear cells; RA, rheumatoid arthritis.
