# Supplementary material for: Activated, Pro-Inflammatory Th1, Th17, and Memory CD4+ T Cells and B Cells Are Involved in Delayed-Type Hypersensitivity Arthritis (DTHA) Inflammation and Paw Swelling in Mice
Source: Front Immunol. 2021 Aug 2;12:689057. doi: 10.3389/fimmu.2021.689057 (PMC8365304; doi:10.3389/fimmu.2021.689057)
Supplement: Supplementary file 1 [file DataSheet_1.docx]

Supplementary Material


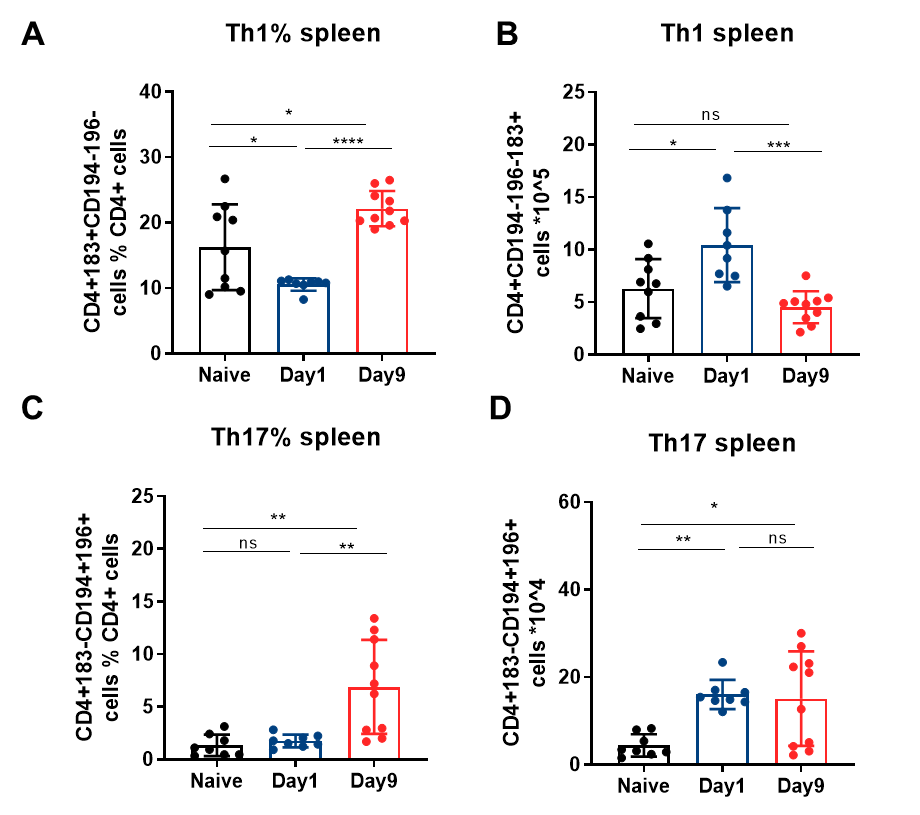


**Supplementary figure 1. Th1 and th17 cell levels in spleen.** Cell suspensions from the spleen of DTHA induced mice and normal on day 1 and 9 post the mBSA challenge were analysed by flow cytometry. (**A and B**). Fraction (%) number (**A**) and absolute number (**B**) of Th1 cells (CD4+CD194-CD196-CD183+) on day 1 and 9 post mBSA challenge. (**C and D**). Fraction (%) number (**C**) and absolute number (**D**) of Th17 cells (CD4+CD194+CD196+CD183-) on day 1 and 9 post mBSA challenge. Data is presented as mean ± SD from two independent experiments with 4-8 mice in each group per experiment with the exception of the data of day 1, which is from one experiment with 8 mice. Statistical significance was calculated by ANOVA with Tukey multiple comparison test (*p<0.05, **p<0.01, ***p<0.001, ****p<0.0001, ns: p>0.05).


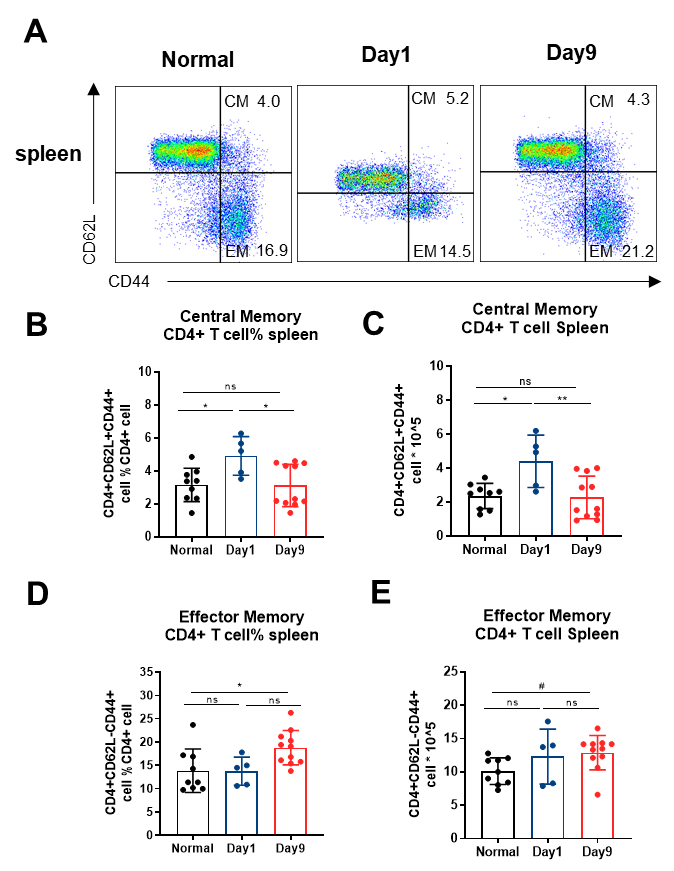


**Supplementary figure 2: Splenic central/effector memory CD4 + T cells in DTHA mice.**

Cell suspension from the spleen of normal mice and DTHA induced mice on day 1 and 9 post mBSA challenge was analysed by flow cytometry. (**A**). Representative flow cytometry dot plot illustrating central/effector memory CD4+ T cells from spleen in normal mice and DTHA induced mice on day 1 and 9 post mBSA challenge. (**B and C**). Relative number (**B**) and absolute number (**C**) of central memory CD4+ T cells (CD4+CD62L+CD44+) from spleen in given groups. (**D and E**). Relative number (**D**) and absolute number (**E**) of effector memory CD4+ T cells (CD4+CD62L-CD44+) from spleen are shown. Data is presented as mean ± SD. Data of normal mice and DTHA induced mice on day 9 are from two independent experiments with at least 5 mice per group per experiment. Data of day 1 group is from one experiment with 5 mice. Statistical significance was calculated by ANOVA with Tukey multiple comparison test (*p<0.05, **p<0.01, ns: p>0.05) or Student’s *t*-test (#: p<0.05). CM: central memory CD4+ T cell. EM: effector memory CD4+ T cell.


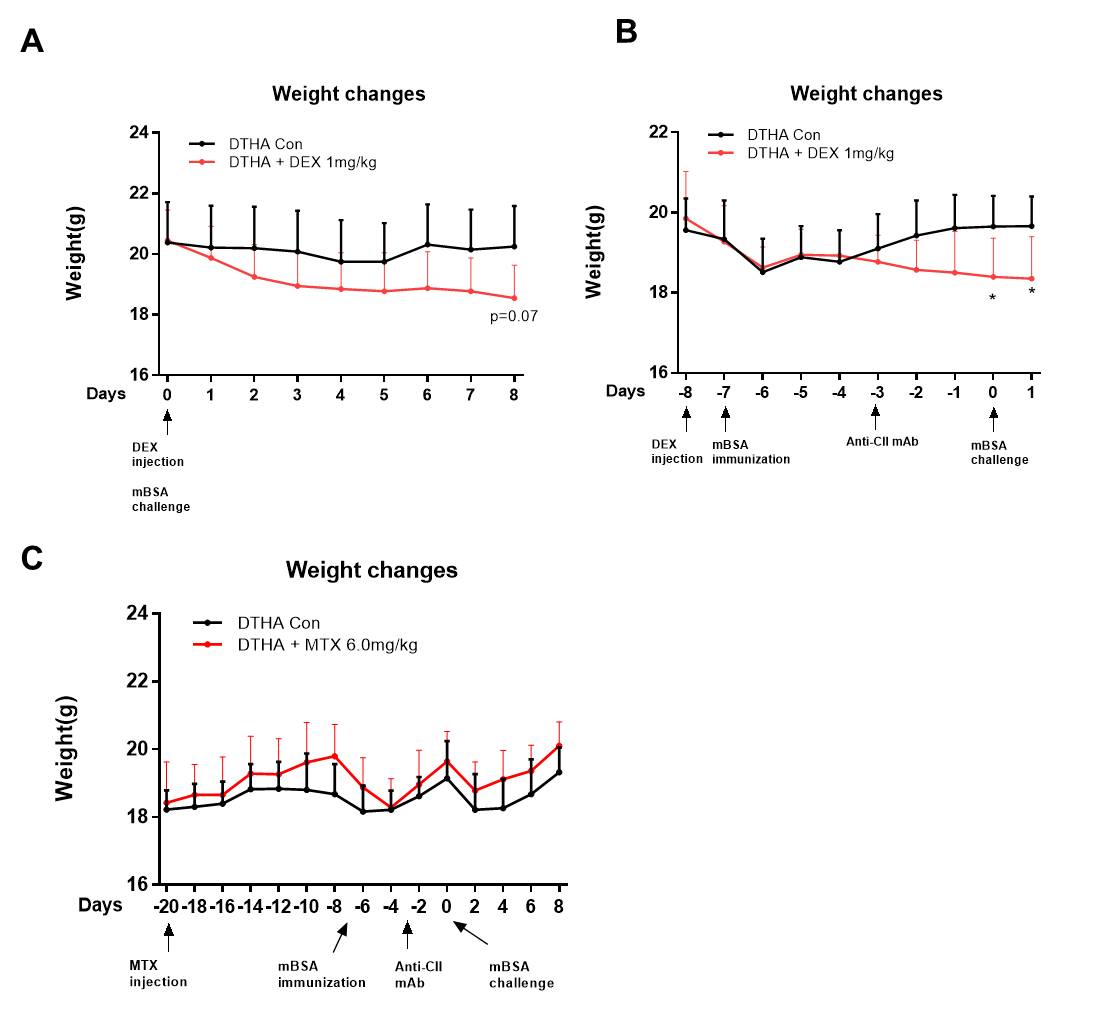


**Supplementary figure 3: Weight changes of mice after treatment of 6mg/kg MTX or 1mg/kg DEX. (A and B).** The weight changes of mice with 1mg/kg DEX administrated every day from the day 0 (**A**) or day -8 (**B**) respectively. (**C**) The weight changes of mice treated with 6mg/kg MTX administrated every other day from the day -20. All data are presented as mean ± SD with 4-8 mice in each group. Statistical significance was calculated by Student’s *t*-test was for the comparison for DTHA control mice and DTHA mice treated with MTX or DEX. P values are listed under the curve if p value < 0.1. (*p<0.05, ns: p>0.05).


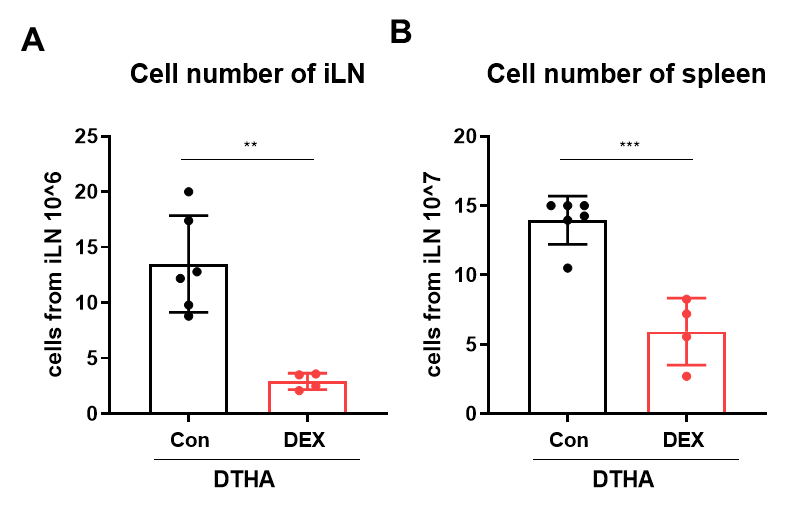


**Supplementary figure 4: Decreased cellularity and cell number of iLN and spleen from mice treated with 1mgDEX/kg. (A and B).** Single cells were obtained and counted by cell counter on day 9 from iLN (**A**) and spleen (**B**) of DTHA mice administrated with or without 1mgDEX/kg every day from day 0. All data are presented as mean ± SD with 4-6 mice in each group. Statistical significance was calculated by Student’s *t*-test was for the comparison for DTHA control mice and DTHA mice treated with DEX. (**p<0.01, ***p<0.001).

**
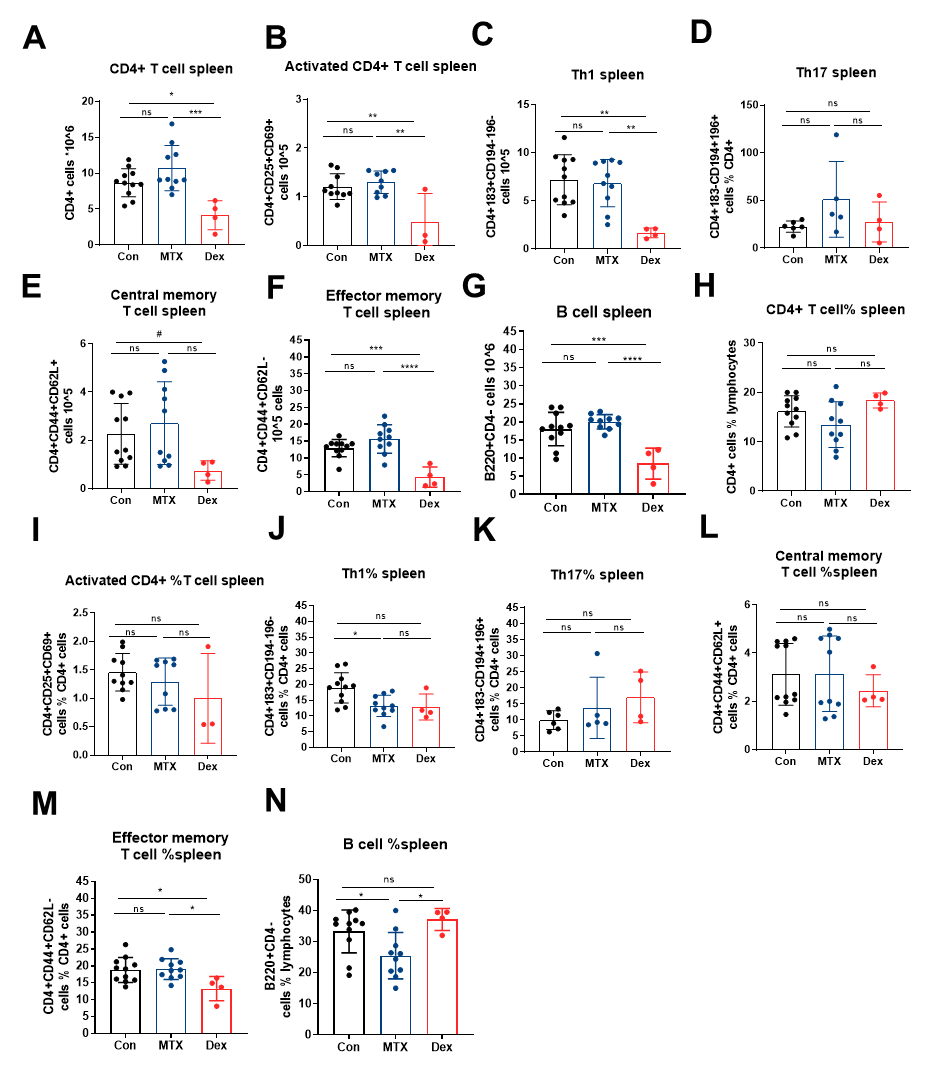
**

**Supplementary figure 5: Differential effects of MTX and DEX on levels of DTHA-induced splenic immune cells.** Cell suspensions from the spleen of DTHA mice without or with MTX or DEX treatment on day 9. Flow cytometry was used to detect CD4+ T cell, activated CD4+ T cell, Th1, Th17, central/effector memory T cell and B cell. The proportion and absolute number of splenic CD4+ T cell (**A and H**), activated CD4+ T cell (**B and I**), Th1 (**C and J**), Th17 (**D and K**), central memory CD4+ T cell (**E and L**), effector memory CD4+ T cell (**F and M**) and B cell (**G and N**) in spleen from mice in given groups are shown. All data are presented as mean ± SD. DEX group data is from one experiment with 4 mice included. All other data are pooled from two independent experiments with 3-6 mice in each group per experiment. Statistical significance was calculated by ANOVA with Tukey multiple comparison test (*p<0.05, **p<0.01, ***p<0.001, ***p<0.0001, ns: p>0.05) or Student’s *t*-test (#: p<0.05).

**Supplementary Figure 6. MTX inhibits mouse CD4+ T cell activation *in vitro*.** Mouse CD4+ T cells were treated with MTX (0-1000nM) overnight followed by stimulation with α-CD3/CD28 beads (1 bead/cell) for 72 hours. At 72 hours cell surface expression of CD69 was quantified by flow cytometry. Statistical significance was calculated by ANOVA with Tukey multiple comparison test (**p<0.01, ****p<0.0001).

| **Antibodies** | **Clone** | **Brand** |
| --- | --- | --- |
| Anti-CD4 | RM4-5 | BD Biosciences |
| Anti-CD25 | PC61 | BD Biosciences |
| Anti-CD69 | H1.2F3 | BD Biosciences |
| Anti-CD62L | MEL-14 | BD Biosciences |
| Anti-CD44 | IM7 | Invitrogen |
| Anti-CD183 | CXCR3-173 | BD Biosciences |
| Anti-CD194 | 2G12 | BioLegend |
| Anti-CD196 | 140706 | BD Biosciences |
| Anti-B220 | RA3-6B2 | BD Biosciences |

**Supplementary table 1. Information of antibodies used for flow cytometry analysis.**
